# Supplementary material for: Are carbon emissions trading and green financial instruments synergistic? -Comprehensive quantitative research based on content analysis
Source: PLoS One. 2024 Mar 7;19(3):e0298601. doi: 10.1371/journal.pone.0298601 (PMC10919601; doi:10.1371/journal.pone.0298601)
Supplement: S1 Data — (ZIP) [file pone.0298601.s001.zip › Data/地级市Z_F工作报告.zip!/╣π╢1⁄2/╖≡╔╜╩╨/└┤╫╘╣1⁄2╓┌║┼í╛┬φ┐╦╩2╛▌═°í┐_2010.docx]

各位代表：

　　现在，我代表佛山市人民政府向大会报告政府工作，请予审议，并请政协各位委员和其他列席人员提出意见。

　　一、2009年工作回顾

　　2009年，是我市进入新世纪以来经济社会发展最为困难的一年。市政府在省委、省政府和市委的正确领导下，在市人大和市政协的监督支持下，坚持以科学发展观统揽全局，坚定不移落实中央、省应对危机的系列政策措施，积极实施《珠江三角洲地区改革发展规划纲要（2008-2020）》（以下简称《规划纲要》），顺利完成市第十三届人大四次会议确定的各项任务。

　　——经济保持平稳较快增长。完成生产总值4814.50亿元，增长13.5%；人均生产总值8.06万元（折合1.18万美元）；地方财政一般预算收入254.70亿元，增长11.71%；税收总额668.75亿元，增长6.25%；全社会固定资产投资1470.56亿元，增长16.8%；社会消费品零售总额1429.05亿元，增长21.3%。

　　——节能环保取得实效。全市单位GDP能耗下降6.93%，主要污染物二氧化硫和化学需氧量排放量分别下降16.12%和12.79 %，完成省下达的年度削减目标，在全省排名第二。城镇污水处理率达68.83%，中心城区生活垃圾无害化处理率达95.8%。

　　——人民生活持续改善。城镇新增就业11.78万人，城镇登记失业率1.84%。城市居民人均可支配收入2.46万元，增长9.3%；农村居民人均纯收入1.07万元，增长10.8％，增幅首次超过城市居民收入。城乡居民储蓄存款余额3945.01亿元，比年初增长12.9%。

　　一年来，我们主要做了以下工作：

　　（一）积极应对国际金融危机，经济在逆势中率先突围

　　抢抓机遇，以重点项目建设拉动经济增长。及时出台促进经济平稳较快发展的政策措施，启动一批重点项目建设，促进了经济企稳回升。全年新增中央投资项目29项，已完成2项，累计完成投资4.53亿元；列入省重点建设项目52项，已立项或开工47项，完成投资236.3亿元，占年度投资计划的106.4%；市重点建设项目289项，已立项或开工266项，预计完成投资360亿元，占年度投资计划的63.8%。

　　内外并举，引领企业开拓市场。落实“家电下乡”政策，开展“广货全国行”等系列活动，引导企业拓展内销市场，累计实现经贸合作金额497.58亿元。稳定外贸出口，引导企业通过展会、经贸洽谈等方式开拓国际市场，共达成贸易协议28亿多美元。全市进出口总值383.39亿美元，其中出口245.78亿美元。

　　加强服务，为中小企业解决发展难题。抓好2800亿元授信额度的落实工作，全年新增贷款总额超过2273亿元，其中企业贷款1312亿元。建立和完善融资担保体系，担保机构共为1880家企业提供融资担保83亿元。南海、顺德区分别实施“雄鹰计划”、“龙腾计划”，有针对性地解决制约中小企业成长发展的劳动力、土地、市场准入等瓶颈问题，促进企业发展壮大。

　　（二）贯彻实施《规划纲要》，区域一体化建设全面推进

　　广佛同城化建设开局良好。与广州市签署合作框架协议，建立市长联席会议制度，出台《广佛同城化发展规划》。以重点项目建设为抓手，共同推动规划、交通基础设施、产业的对接以及水和大气污染的联防联治。去年计划推进的52个重点项目有46项进展顺利，其中15项已完成。

　　融入珠三角一体化发展初见成效。配合做好珠三角基础设施、产业发展、环境保护、城乡规划和公共服务五个一体化专项规划。推动与珠三角高速公路、轨道交通互联互通。广肇城际轨道佛山至肇庆段动工建设，广珠西二期、江肇等高速公路建设顺利。启动广佛肇经济圈建设，三市确定首批37个重点合作项目。

　　佛港澳深化合作取得突破。出台落实CEPA示范城市实施方案，与港澳建立政府高层沟通互动机制。佛港澳确定在科技业、先进制造业、现代服务业、社会事务业等4个方面开展重点交流合作。佛港在金融、运输、教育培训、医疗门诊、工业设计、粤港电子签名证书互认试点应用等领域合作进展良好，4家港资银行获批在佛山设立异地支行。成功承办全国商务系统落实CEPA示范城市佛山现场工作会。

　　（三）大力推进产业转型，现代产业体系建设初见成效

　　现代农业向“精、优、强”发展。规划建设现代农业园区26个，其中省级园区２个。国通物流、海峡两岸创意农业城、兰花科技园等23个重点项目建设顺利推进。农业产业化、组织化水平得到提高，农业标准化、品牌化建设取得新成绩，顺德区成为“中国鳗鱼之乡”，三水区“乐平雪梨瓜”获评国家地理标志保护产品。

　　工业结构调整优化成效明显。全社会工业总产值12743.49亿元，增长15.1%。全市高新技术产品产值2600亿元，增长20%，新增高新技术企业153家。光电显示、新材料等一批新兴产业加快发展，我市列为首批国家新型工业化产业示范基地。以招商引资优化产业结构，全年新增世界500强项目8个，全市合同利用外资10.13亿美元，实际利用外资18.74亿美元。以高新技术和信息化改造提升传统产业，我市成为国家级信息化和工业化融合试验区。加快淘汰落后产能，关停一批高耗能高污染企业。“双转移”工作取得新成绩，佛山（清远）、（云浮）产业转移园均成为省级示范转移园区。

　　服务业发展实现新突破。第三产业实现增加值1689.99亿元，增长16.1%，占GDP比重35.1%，同比提高1.2 个百分点。广东金融高新技术服务区建设加快，累计引进23个项目，投资总额65亿元。举办佛山首届现代服务业暨第六届佛山（国际）物流合作洽谈会，我市成为全国流通领域物流示范城市，顺德区乐从镇获批首个国家级电子商务试点。举办首届“中国（佛山）陶瓷节”和机械装备、节能环保、家电、家具等专业展会。广东工业设计城引进50多家国内外著名企业，佛山创意产业园、南海动漫虚拟创业园建设初具规模。

　　自主创新能力得到提高。积极开展与中科院的战略合作，共建中科院佛山产业技术创新与育成中心，促成各类院企合作项目近100项。禅城区成为国家科技进步示范区。公共技术服务平台建设加强，设立佛山火炬创新创业园等9个科技企业孵化器，在孵高新技术企业320家，新增省级工程技术研究开发中心8家。专利、品牌、标准建设取得成效，全市发明专利申请量和授权量分别增长17.7%和76.5%，新增中国驰名商标9件，参与制定（修订）国家标准105项、行业标准8项。人才战略有效实施，引进外国专家及留学人才450名。

　　（四）大力推进城市转型，城市现代化和综合竞争力不断提高

　　统筹城乡发展迈出新步伐。制定并实施统筹城乡发展的系列政策文件，启动首批28个统筹城乡发展重点项目、5个宅基地换房试点工作。13个村居完成集体经济体制改革试点任务，2467个村组完成“两确权”工作，南海区基本解决“出嫁女”权益问题。集体土地和财务管理进一步规范，三水区率先建立集体土地经营权流转中心和开展消除农村财务“白头单”结算试点。镇（街道）公交不断完善，全市行政村公交通达率、行政村之间道路硬底化率均达100％。

　　“三旧”改造成效显著。出台《佛山市三旧改造专项规划（2009－2020年）》，积极争取国家、省的政策支持。全市启动改造项目229个，完成改造项目115个，完成改造新建面积710万平方米。祖庙-东华里、高明区旧汽车站、三水区西南涌等一批改造项目成效显著。成功承办全省“三旧”改造工作现场会。

　　重点基础设施建设加快推进。广佛轨道交通首通段土建施工基本完成。广明高速西樵至更楼段、广佛高速扩建、西二环和顺立交收费站及连接线建成通车。禅西大道一期、“一环”南拓一期等33个市重点项目动工建设。佛山机场民航成功复航。第二饮用水源首期工程试产通水。“U-佛山”建设全面铺开，一批重点信息化项目建设进展顺利。

　　城市建设和管理不断加强。各城市组团规划进一步完善。东平新城启动公共文化综合体和区域性交通枢纽站等11个重点项目建设。实施“大城管”、“大综管”战略，城市综合执法和综合监管力度加大，城市环境和秩序明显改善。积极打造“数字城管”，市级数字化城市管理信息系统项目开工建设。

　　（五）大力推进环境再造，科学发展的支撑体系日趋完善

　　生态环境建设取得新成绩。新建、续建、扩建污水处理厂24间，新增污水处理能力67.3万吨/日。以汾江河及内河涌为重点的水环境综合整治成效显著，时隔26年成功举行端午节汾江河龙舟赛。以机动车尾气、陶瓷行业污染物排放为重点的大气污染源得到有效治理，全市空气质量持续好转。“三年促变，绿地佛山”战略深入实施，新增绿地面积203万平方米，改造绿地面积567万平方米。顺利完成国家园林城市复查迎检工作。

　　体制机制改革实现新突破。市级政府机构改革全面完成。顺德区开展综合改革试验工作，并实施大部制改革，市级部门进一步向顺德区下放行政审批和日常管理事项。南海区狮山镇、顺德区容桂街道简政强镇事权改革试点顺利推进。行政审批流程改革取得成效，我市被省政府确定为创新行政审批方式试点城市。事业单位分类改革稳步推进。金融发展三项计划成果显著，5家小额贷款公司获准开业，新增上市公司4家，南方风机成为全国首批创业板上市企业；知识产权质押、企业股权质押等新型融资方式得到推广；顺德农信社成功改制为农商行并挂牌营业。联合国支持产业集群与资本运作（佛山）国际示范城市项目正式运作。保险业服务“三农”取得实效，全市政策性农村住房保险投保覆盖率超过61%，三水区建立国内首例“政银保”合作农业贷款模式。财政体制改革继续深化，探索财政资金竞争性分配改革试点，将绩效评价引入预算资金分配。国库集中支付制度改革加快，公务卡改革全面实施。

　　社会事业发展取得新进步。全市财政一般预算公共服务和民生事业支出236.98亿元，增长9.62%。就业和社会保障工作继续加强，通过开发、收集公益性岗位安置城乡就业难人员，通过举办各类招聘会促进高校毕业生和农民工就业。城乡一体化养老保障体系不断完善，南海区试点将符合条件的城乡居民、顺德区将完全被征土地农村居民纳入城镇职工社会保障体系。启动新型农村社会养老保险工作。教育现代化建设稳步推进，禅城、南海、顺德区被评为“广东省推进教育现代化先进区”。推进全市中小学校舍安全工程，做好中小学代课教师入编工作。医疗卫生服务体系不断健全，社区、村居卫生服务网络日趋完善，甲型H1N1流感得到有效防控。继续稳定低生育水平，我市人口自然增长率为5.02％。。文化事业蓬勃发展，成功举行“魅力佛山·秋色辉煌”大巡游及第八届亚洲游泳锦标赛等一批体育赛事，我市荣获首个“中国龙狮龙舟运动名城”称号。社会治安综合治理力度加大，南海区率先建立镇级综治信访维稳中心。安全生产专项整治，农产品、食品、药品质量监管进一步加强。人民调解、信访工作、环境污染、公共安全等突发事件应急机制进一步健全。支援汶川水磨镇重建工作扎实有效，“规划到户，责任到人”扶贫开发工作积极推进。

　　十件民生实事顺利完成。全市政策性农业保险水稻险种实现全投保，覆盖率为100%。建设保障性住房2440套。解决了175户人均住房面积不到12平方米的渔民住房问题。新建45间社区卫生服务站，完成205间社区卫生服务站信息化基础设施配套建设。居民住院医保实现市级统筹，门诊基本医保报销范围扩大；大学生及中职校学生被纳入居民医保对象。开展生猪及其肉品专项整治行动，蔬菜抽检合格率超过99%，生猪瘦肉精抽检合格率达99.8%，生鲜乳三聚氰胺抽检合格率达100%。全市完成200人以上、革命老区50人以上自然村农村公路建设项目12个。完成老区村饮水改造工程132个。全年农村（社区）放映公益电影1.12万场次，引进高雅艺术展演17场。企业退休人员月人均养老金提高到1277元。禅城、南海、顺德区低保标准提高到350元/人·月，高明、三水区提高到310元/人·月。

　　政府自身建设进一步加强。积极向人大常委会报告依法行政工作情况，自觉接受人大的法律监督、工作监督和政协的民主监督。办理人大代表建议和政协提案282件，办复率达100%。全面清理政府及其部门的规范性文件，主动公开政府信息，政府公信力和透明度得到提高。积极推行网络问政，政府科学、民主、依法决策进一步完善。认真落实党风廉政建设责任制，强化反腐倡廉各项工作。厉行节约，大力压缩行政开支。开展以“廉政、效能、服务”为主题的机关作风建设活动，政风行风进一步好转。

　　与此同时，老年人、残疾人、妇女儿童事业，双拥优抚、粮食储备、物价、统计、民族宗教、外事侨务、对台事务、档案、方志、人防、气象等各项工作都取得了新成绩。

　　各位代表！回顾一年来的工作，我们获得以下几点体会：一是必须坚持解放思想、与时俱进，正确研判形势，准确把握发展规律和趋势，不断提高科学决策和领导科学发展的能力。二是必须坚持民营企业是佛山发展之根本，同时充分认识利用外资促进经济发展的重要作用，大力实施“双轮驱动”战略，推动内外源型经济协调发展。三是必须坚持扩大投资规模和优化投资结构并举，大力实施重点项目带动战略，推动经济平稳较快增长。四是必须坚持发展经济与改善民生、维护社会公平正义的内在统一，统筹城乡一体化发展，缩小城乡二元差距，实现城乡共同繁荣进步。五是必须坚持科学发展、先行先试，以改革创新精神，加强政府自身改革和建设，更好地发挥推动科学发展、促进社会和谐稳定的引领作用。

　　各位代表！过去一年我们经受了严峻考验，取得了来之不易的成绩，这是省委、省政府和市委正确领导的结果，是全市人民团结一心、共同奋斗的结果。在此，我代表佛山市人民政府向全市广大干部群众，中央、省驻佛山单位，驻佛山人民解放军指战员、武警官兵和社会各界人士表示崇高的敬意，向长期关心支持佛山建设的港澳台同胞、海外侨胞、国内外友人以及外来务工人员表示衷心的感谢！

　　我们也清醒地认识到，我市经济社会发展仍然存在一些矛盾和问题，主要是：自主创新能力不强，产业转型升级任务繁重，提升服务业发展水平力度亟待加大；土地、能源、环境约束日益突出，加快转变经济发展方式任重道远；统筹城乡一体化发展仍需加强；城市生活环境和城市国际化水平有待提高；社会和谐稳定还存在较大隐忧；政府执行力有待进一步加强，等等。我们要正视这些问题，并在今后工作中认真加以解决。

　　二、2010年工作总体要求和目标任务

　　今年是继续应对国际金融危机、保持经济平稳较快发展、加快转变经济发展方式的关键一年，是全面实现“十一五”规划目标、为“十二五”发展打好基础的重要一年，也是深入贯彻落实《规划纲要》并力争取得重大成效的一年。

　　做好今年的政府工作，要以科学发展观为指导，认真贯彻落实党的十七届四中全会、中央经济工作会议、胡锦涛总书记视察广东重要讲话精神，以及省委十届六次全会和市委十届八次、九次全会精神，深入实施《规划纲要》，以“四化融合，智慧佛山”为目标，以转变经济发展方式为核心，大力实施“三着力一推进”战略部署，坚定不移调结构，脚踏实地促转变，全面完成“十一五”规划各项目标任务，努力实现经济社会又好又快发展。

　　今年经济社会发展主要预期目标是：生产总值增长12%；人均生产总值增长11%；单位GDP能耗完成省下达任务；主要污染物二氧化硫和化学需氧量排放量均完成省下达任务；全社会固定资产投资增长16%；进出口总额增长5%；社会消费品零售总额增长18%；地方财政一般预算收入增长10%；服务业增加值占GDP比重达36%；全社会试验研究与试验发展（R&D）投入占GDP比重达1.8%；城市居民人均可支配收入增长8.5%；农村居民人均纯收入增长9%；居民消费价格总水平涨幅控制在3%以内；城镇登记失业率控制在4%以内；人口自然增长率控制在6.5％。以内。

　　为实现上述目标任务，我们要重点抓好四个方面工作：

　　（一）着力转变经济发展方式，加快构建现代产业体系

　　积极扩内需调结构。进一步落实家电、汽车下乡及“以旧换新”等促进消费的政策措施，继续推动企业参加国内各类产品展销会和开展“广货全国行”活动。加强房地产市场调控，探索建立政策性租赁住房制度，加快以廉租房为主的保障性住房建设。加强和改进投资管理，切实抓好一批重点项目的申报、立项和动工建设。拓宽民间投资领域，引导各类资本投向重大基础设施和产业项目。完善社会保障体系，稳步提高城乡居民收入，增强城乡居民特别是低收入群众消费能力。进一步优化出口产品结构，大力发展服务贸易和服务外包业，积极推进加工贸易转型升级。继续实施“走出去”战略，积极应对国际贸易摩擦。

　　大力发展战略性新兴产业。落实我市发展战略性新兴产业的工作方案和扶持办法，以建设国家新型工业化产业示范基地为契机，统筹发展战略性新兴产业。大力发展光电、新材料、现代服务业、新医药、环保、新能源汽车等六大产业，其中重点主攻光电、新材料、现代服务业，力争到2012年这三大产业规模均超1千亿元。加大招商引资力度，着力引进先进制造业、战略性新兴产业和现代服务业。

　　加快发展现代服务业。积极实施服务业“314”计划［1］，稳步提高服务业发展水平和在国民经济中的比重。坚持生产性服务业与生活性服务业发展并举，完善促进服务业发展的政策环境和扶持措施，鼓励各类社会资本进入。重点发展金融服务、现代物流、商务会展、创意设计、信息服务、总部经济等十一类服务业。推动服务业产业链条不断延伸，促进服务外包业发展。进一步推进佛港澳在科技业、现代服务业等领域的深入合作，抓紧落实佛港、佛澳合作各项重点项目。加快推进一批服务业重点项目建设，抓好“广东九江综合保税区”创建工作，支持顺德区申报国家服务业综合改革试点。

　　继续推进传统产业转型升级。加快实施我市优势传统产业转型升级行动方案，引导企业运用信息技术、科技创新、商业模式创新、价值链提升等手段改造提升传统产业。以联合国支持产业集群和资本运作（佛山）国际示范城市项目建设，带动优势传统产业转型升级；以发展先进制造业引领传统产业转型升级。打好节能减排攻坚战和持久战，进一步淘汰落后产能，促使企业走低碳、清洁、可持续发展道路。继续做好“双转移”工作。落实民营经济发展政策，加强和改善政府服务，引导和支持民营企业通过股份制改造、资本市场上市、技术创新、品牌培育等手段加快向规模化、集约化、品牌化、国际化方向发展壮大。

　　以“四化融合”构建“智慧佛山”。认真实施《四化融合，智慧佛山发展规划纲要（2010-2015）》，推动信息化、工业化、城市化、国际化的融合。以信息化带动工业化，既要培育与信息化相关联的新兴产业，又要运用信息化手段改造提升传统产业，深化信息技术在传统产业各个环节的运用和渗透。以信息化提升城市化，既要积极推进电信网、电视网、互联网三网融合，加快物联网发展，为人民群众的生产、生活提供便捷高效的信息服务，又要借助信息化手段和措施，大力发展智能交通、智能治安、智能教育、智能医疗、智能文化等等，使我市加快迈向信息社会。以信息化加快国际化步伐，既要在微观层面引领企业不断应用信息化手段开拓国际市场，参与国际合作与竞争，又要在宏观管理上加快实施通关电子化、一体化，为企业走向世界提供更优质的服务，实现国际化发展。深化佛港澳电子商务应用合作，大力发展信息服务外包业，打造广东重要的外包服务业基地。

　　加强自主创新能力建设。全面推进与中科院的战略合作，加紧建设中科院佛山产业技术创新与育成中心，促进先进科技成果在佛山转化。加快制定知识产权战略规划纲要，实施专利、标准、品牌战略工作考核办法，引导和支持企业建立专利联盟、标准联盟和品牌联盟。加强区域科技创新体系建设，培育发展风险投资、技术交易、公共检测等中介服务机构，推动一批重点产业创新平台、博士后工作站、院士工作室落户佛山。积极争取广东金融高新区成为“新三板”试点园区。实施优秀人才引进培养工程，着力引进海内外高层次创新型人才来我市创业和发展。

　　（二）着力实施农村居民收入倍增计划，加快城乡一体化发展

　　千方百计促农村居民增收。实施农村居民收入倍增计划，通过物业改造、转移就业、合作经营、农民创业、财税补贴等多种途径，提高农村居民的财产性、工资性、经营性、转移性收入，逐步缩小城乡居民收入差距。支持高明区加快革命老区脱贫致富步伐。

　　进一步激发农村改革活力。全面铺开农村集体经济体制改革试点，加紧完成“两确权”工作，推进“股权到人改股权到户”改革，有效解决“出嫁女”权益问题。稳步开展农村产权制度改革试点。加快农村宅基地换房试点建设，完成首批试点村居建设任务。探索开展城乡建设用地增减挂钩工作试点。全面推进集体林权制度改革。 推动城市基础设施向农村延伸。编制《佛山市城乡统筹规划》，以规划引领城市基础设施向农村延伸。重点加快公路网、交通站场、天然气管网、生态建设等9大类城市基础设施向农村延伸项目的启动和建设，力争到今年底，全市城市化水平达92.37%，自然村通公交率达70%，城镇污水处理率达77%。

　　推进基本公共服务均等化。制定基本公共服务均等化实施细则和绩效考评机制，探索建立多元化的公共服务供给制度。以农村社区建设为平台，探索在村（居）建立行政服务中心，将公共管理和服务职能延伸到乡村。加快农村各项社会事业发展，逐步缩小城乡、区域之间的基本公共服务差距。

　　以园区建设引领现代农业发展。全面推进现代农业园区建设，提高农业综合生产能力。加强农业园区招商引资，培育引进一批辐射带动能力强的现代农业项目，推动农业龙头企业上市融资。加快农业科技创新和信息技术推广应用，提高农产品的科技含量和附加值，提升农业产业化经营水平，增强农业核心竞争力。

　　以“三旧”改造重塑城乡新貌。实施“三旧”改造“532”工程［2］，用3年时间实现“旧貌换新颜，三年建造新佛山”目标。今年争取完成“三旧”改造项目用地面积5万亩，并培植30个市级先进改造示范点。用足用好“三旧”改造政策，完善历史用地手续。加强土地储备，增强政府土地调控能力。

　　（三）着力提升民生社会事业，加快建设富裕和谐佛山

　　以“迎亚运”为契机，全力维护佛山安全和谐稳定。扎实做好亚运佛山赛区安保工作。完善社会治安预警机制和防控体系，依法坚决打击涉黑势力、团伙犯罪以及“两抢一盗”等各类违法犯罪活动，推进“平安佛山”建设。加强信访维稳和人民调解工作，扎实做好法律服务、社区矫正和帮教安置工作。全面实施居住证制度，加强和改善流动人口管理和服务。进一步加强应急平台建设。落实企业安全生产主体责任，加强安全生产隐患排查治理工作，强化食品、药品、农产品安全质量监管，坚决遏制重特大事故发生，保障人民群众生命财产安全。

　　以完善路网系统为核心，大力加强现代化综合交通体系建设。落实城乡交通升级计划及实施方案，统筹区域交通站场、港航建设，促进各种交通运输方式“零换乘”和无缝对接。加快广佛轨道交通建设，确保首通段今年10月建成通车。启动广佛环线建设。加快推进禅西大道、“一环”西延线、广明高速东段等重点项目建设。稳步推进中心城区限摩工作。继续实施公共交通共同体模式改革，提升公交服务水平，力争今年实现全市交通公交分担率12％的目标。加强城市规划、建设和管理。积极创建全国无障碍建设城市，力争今年通过国家验收。加快“数字城管”建设，利用信息技术优化城市资源配置，提升城市管理水平。

　　以建设宜居城乡为目标，大力加强环境保护和资源节约利用。实施迎亚运环境升级计划，进一步强化工业污染、机动车尾气、挥发性有机气体污染的控制和整治。全面开展跨界河流、广佛接壤区域河涌和比赛场馆周边河涌的治理和保洁工作。大力推进节能、节水、节地、节材和资源综合利用，加强饮用水源保护。全面完成城乡水利防灾减灾工程建设任务。

　　严格落实节能减排问责制，确保完成“十一五”节能减排目标任务；严格执行最严格的耕地保护制度，坚决查处违规违法用地行为。积极创建“全国绿化模范城市”。全面推进绿道[3]规划建设，构建城市道路绿网和水系绿网骨架，建设岭南绿城。加强“不开发区”规划建设，建立基本农田保护补贴等生态补偿机制。

　　以保障和改善民生为重点，大力发展社会各项事业。继续实施更加积极的就业政策，重点做好城乡就业难人员、高校毕业生、农民工的就业服务工作，鼓励和支持自主创业和自谋职业，以创业带动就业。加强人文关怀，改善用工环境。进一步完善城乡一体化的社会保障体系，探索农民工参加社会养老保险办法，鼓励农村居民参加企业职工基本养老保险。促进教育事业均衡优质发展。加快教育现代化建设，努力争创国家教育综合改革试验区。加快佛山科学技术学院、佛山职业技术学院、佛山高级技工学校规划建设。落实教师平均工资水平“两相当”[4]。深化医药卫生体制改革，完善基本医疗保障制度，逐步建立基本药物制度，健全基层医疗卫生服务体系。稳妥开展公立医院改革试点。加强公共卫生服务体系建设，切实做好重大传染病和职业病防控工作。认真落实人口计生政策，有效控制人口增长，提高人口素质。全面推进全国文明城市创建活动，深入开展“情暖佛山”系列活动，不断提高市民文明素质和现代化大城市意识。大力发展文化事业，加强历史遗址、非物质文化遗产的保护和开发利用，全面完成祖庙修缮工作。推进文化资源整合，把“佛山秋色欢乐节”打造成为佛山特色、中国知名的文化品牌。积极创建“中国书法名城”。加强城市形象宣传，打造城市品牌，提升佛山知名度。贯彻落实《全民健身条例》，加快形成崇尚健身的良好社会风气。全力办好亚运会拳击和花样游泳比赛。全面落实双拥优抚安置政策，争创“全国双拥模范城市”。积极推动广佛同城化和广佛肇经济圈建设，让同城化和区域一体化成果更好地惠及佛山市民。

　　我们要坚决贯彻中央、省委关于对口援疆、扶贫开发工作的决策和部署。积极开展对口支援新疆伽师县工作，力争年内初见成效。继续推进“规划到户、责任到人”扶贫开发，通过智力扶贫、劳务扶贫等方式，提高帮扶地区“造血”功能，达到自主脱贫。全面完成支援汶川水磨镇恢复重建任务。

　　重点办好10件民生实事。（1）加强保障性住房建设。今年计划建设保障性住房1600套，力争到年底基本解决2007年调查在册的7400户、人均居住面积12平方米以下低收入家庭的住房问题。（2）全面实施新型农村养老保险制度。建立覆盖全体农村居民、城乡统筹的养老保险制度。（3）实施基本农田保护补贴。将全市基本农田列入“不开发区”范围，依法严格保护，对承担基本农田保护任务的农村集体经济组织或其他责任单位给予财政补贴。（4）实施政策性农业保险。全面推行政策性农村住房保险，在顺德区开展政策性生猪保险险种试点。（5）加强公共卫生服务能力建设。常住人口基本公共卫生服务经费标准达到人均25元以上。新建24间社区卫生服务站。启动市第三人民医院住院综合大楼建设和第四人民医院改造工程。（6）实施中小学校舍安全工程。今年全市要按省要求的校舍安全工程改造项目，完成加固、改造、重建校舍工程9.51万平方米。（7）扩大“明天计划”实施范围。将“明天计划”扩大到159名低保临界家庭残疾子女，安排适宜手术的122名低保临界家庭儿童入院实施手术。（8）实施农村特困家庭危房改造。对270户农村低保户、散居五保户等困难群众危旧房进行改造。（9）完善高龄老人津贴制度。从今年7月1日起，对全市90周岁以上户籍老人发放高龄津贴。其中，90~99岁老人每人每月150元，100岁以上老人每人每月300元。（10）提高企业退休人员基本养老金。从今年1月起，对2009年12月31日前（含本日），已领取基本养老金的23.46万多名企业退休人员调整基本养老金。

　　（四）推进改革促发展

　　深化行政管理体制改革。参照顺德区大部制改革模式，在全市各区深入推进党政机构改革。支持顺德区深入开展综合改革试验工作。在全市各镇（街道）全面开展简政强镇事权改革，强化面向基层和群众的社会管理和公共服务职能。进一步完善和深化事权下放工作，重点做好区向镇（街道）下放事权工作。加强行政审批电子网络一体化建设，将行政服务内联五区、外延村居。稳步推进事业单位分类改革和人事制度改革。

　　加快社会管理体制改革。探索推进公共服务市场化改革，建立健全政府购买公共服务机制。积极倡导和支持志愿者服务，培育发展行业协会、商会、社团、公益慈善机构等非营利性社会组织，引导社会组织诚信自律、良性发展。加强城乡社区建设，为明年村（居）换届选举工作打好基础。深化户籍制度改革，建立农民工积分制入户制度，逐步使优秀农民工融入城市。

　　深化财政金融体制改革。坚持公共财政原则，优化财政支出结构，加大对民生领域和社会事业的投入。建立和完善公共财政预算编制、执行、监督与问效体系。加强公共融资平台监管制度建设，防范政府债务风险。深入实施金融发展三项计划，大力推进金融业创新发展，争取成为中小企业融资改革综合试点区。加快农村金融服务体系建设，稳步推进农信社分批改制和村镇银行试点工作，推广政策性农业贷款担保基金模式。大力发展资本市场，推动更多企业上市融资，打造证券市场“佛山板块”。

　　各位代表！全面完成今年各项任务，必须进一步加强政府自身改革和建设。深入推进依法行政，严格规范权力运行。自觉接受市人大及其常委会的法律监督和工作监督，主动接受市政协的民主监督，重视群众监督和舆论监督。加快政府职能转变，进一步推进政府科学决策、民主决策、依法决策。科学编制好“十二五”国民经济和社会发展规划。加强电子政务建设，完善网络问政平台，深入推进政务信息公开。加强政风行风建设，建立政府绩效考评体系，提高行政效能。全面落实行政执法责任制，完善行政问责制度。严格执行党风廉政建设责任制，加快推进惩治和预防腐败体系建设，坚决查处各种违纪违法案件。继续推进节约型机关建设，强化公务员队伍教育、管理和监督，努力建设一支为民务实清廉的公务员队伍。

　　各位代表！佛山正处于经济转型、社会转轨的关键时期，困难和挑战考验着我们，责任和使命激励着我们。让我们在省委、省政府和市委的正确领导下，团结和依靠全市人民，迎难而上，奋力拼搏，为续写佛山发展新辉煌而努力奋斗！
